# Excitatory and inhibitory hippocampal neurons differ in their homeostatic adaptation to chronic M-channel modulation

**DOI:** 10.3389/fnmol.2022.972023

**Published:** 2022-10-14

**Authors:** Lior Bar, Lia Shalom, Jonathan Lezmy, Asher Peretz, Bernard Attali

**Affiliations:** Department of Physiology and Pharmacology, Sackler Faculty of Medicine, Sagol School of Neuroscience, Tel-Aviv University, Tel Aviv, Israel

**Keywords:** axon initial segment, homeostatic plasticity, M-channels, potassium channel, synaptic plasticity

## Abstract

A large body of studies has investigated bidirectional homeostatic plasticity both *in vitro* and *in vivo* using numerous pharmacological manipulations of activity or behavioral paradigms. However, these experiments rarely explored in the same cellular system the bidirectionality of the plasticity and simultaneously on excitatory and inhibitory neurons. M-channels are voltage-gated potassium channels that play a crucial role in regulating neuronal excitability and plasticity. In cultured hippocampal excitatory neurons, we previously showed that chronic exposure to the M-channel blocker XE991 leads to adaptative compensations, thereby triggering at different timescales intrinsic and synaptic homeostatic plasticity. This plastic adaptation barely occurs in hippocampal inhibitory neurons. In this study, we examined whether this homeostatic plasticity induced by M-channel inhibition was bidirectional by investigating the acute and chronic effects of the M-channel opener retigabine on hippocampal neuronal excitability. Acute retigabine exposure decreased excitability in both excitatory and inhibitory neurons. Chronic retigabine treatment triggered in excitatory neurons homeostatic adaptation of the threshold current and spontaneous firing rate at a time scale of 4–24 h. These plastic changes were accompanied by a substantial decrease in the M-current density and by a small, though significant, proximal relocation of Kv7.3-FGF14 segment along the axon initial segment. Thus, bidirectional homeostatic changes were observed in excitatory neurons though not symmetric in kinetics and mechanisms. Contrastingly, in inhibitory neurons, the compensatory changes in intrinsic excitability barely occurred after 48 h, while no homeostatic normalization of the spontaneous firing rate was observed. Our results indicate that excitatory and inhibitory hippocampal neurons differ in their adaptation to chronic alterations in neuronal excitability induced by M-channel bidirectional modulation.

## Introduction

Neural circuits preserve the delicate balance between stability and adaptability, by using various mechanisms of homeostatic plasticity to stabilize firing rates at a certain set-point in response to bidirectional perturbations of neuronal activity (Turrigiano et al., 1998; Abbott and Nelson, 2000; Burrone et al., 2002; Desai et al., 2002; Turrigiano and Nelson, 2004; Davis, 2006; Marder and Goaillard, 2006; Turrigiano, 2011; Slomowitz et al., 2015; Vertkin et al., 2015; Styr and Slutsky, 2018). These compensatory changes include modulation of both intrinsic excitability and synaptic function. The homeostatic regulation of intrinsic excitability involves alterations in ion channels expression and in the structural and functional characteristics of the axon initial segment (AIS) (Turrigiano, 2011, 2012; Yoshimura and Rasband, 2014; Kole and Brette, 2018). The homeostatic synaptic plasticity includes modifications of synapse number and structure, synaptic strength, excitation-to-inhibition (E/I) balance *via* the regulation of postsynaptic neurotransmitter receptors or the regulation of the readily releasable pool of synaptic vesicles (Beattie et al., 2002; Thiagarajan et al., 2005; Wierenga et al., 2005; Stellwagen and Malenka, 2006; Sutton et al., 2006; Hou et al., 2008; Ibata et al., 2008; Jakawich et al., 2010; Kim and Ryan, 2010; Turrigiano, 2012; Yin and Yuan, 2014; Wefelmeyer et al., 2016).

Network firing arises from the complex interplay between synaptic and intrinsic neuronal properties; however, little is known about the interaction between synaptic and intrinsic homeostatic plasticity. Many lines of evidence indicate that neural networks use different types of regulatory mechanisms to achieve homeostasis over a wide range of temporal and spatial scales (Turrigiano and Nelson, 2004; Davis, 2006; Marder and Goaillard, 2006; Turrigiano, 2011, 2017; Styr and Slutsky, 2018). Homeostatic synaptic scaling and modulation of intrinsic excitability regulate firing rates around a set point value. Yet, it is not clear whether these two forms of homeostasis are redundant or are induced to perform distinct functions, or whether these two processes are interacting to achieve complementary tasks. Recently, we showed that chronic neuronal hyperactivity, induced by M-channel inhibition, triggered intrinsic and synaptic homeostatic plasticity at different timescales in cultured hippocampal excitatory neurons (Lezmy et al., 2020). Homeostatic adaptation of intrinsic excitability occurred at a fast timescale (1–4 h) including changes in the threshold current and a distal relocation of FGF14, which physically bridges Nav1.6 and Kv7.2/3 channels along the axon initial segment (AIS) (Pablo and Pitt, 2017; Lezmy et al., 2020). In contrast, synaptic homeostasis occurred at slower timescale (≈ 2 days) and involved decreases in miniature excitatory post-synaptic current (mEPSC) amplitude (Lezmy et al., 2020). However, the fast intrinsic adaptation of excitatory neurons was not sufficient to account for the slow homeostatic normalization of the mean firing rate (MFR). In contrast, homeostatic adaptation of intrinsic excitability and spontaneous MFR failed in hippocampal GABAergic inhibitory neurons, which remained hyperexcitable following chronic M-channel blockage (Lezmy et al., 2020).

Here, we examined whether the homeostatic plasticity induced by M-channel inhibition was bidirectional by investigating the acute and chronic effects of the M-channel opener retigabine (RTG) on hippocampal neuronal excitability. While acute RTG exposure decreased excitability in both excitatory and inhibitory neurons, chronic RTG treatment triggered in excitatory neurons homeostatic adaptations at a time scale of 4–24 h, which was accompanied by a significant decrease in the M-current density and a small proximal relocation of Kv7.3 and FGF14 segments in the AIS. In inhibitory neurons, no homeostasis of the MFR occurred, while compensatory changes in intrinsic excitability were hardly observed and only after 48 h. Thus, long-lasting M-channel bidirectional modulation in the hippocampus can trigger in excitatory but not inhibitory neurons, a homeostatic plasticity though with somewhat different kinetics and mechanisms.

## Materials and methods

### Animals

Balb/c mice of either sex were used for generating the primary cultures of hippocampal neurons. All experimental protocols conformed to the guidelines of the Institutional Animal Care and Use Committee of Tel-Aviv University, Israel, and to the guidelines of the NIH (animal welfare authorization number 01-16-012).

### Drugs

Retigabine dihydrochloride (Alomone R-101), XE991 dihydrochloride (Tocris 2000/10), Bicuculline Methiodide (Alomone B-136), D-2-Amino-5-phosphonovaleric acid (AP-5, Alomone D-145), NBQX disodium salt (Alomone N-186), Tetrodotoxin citrate (Alomone T-550), 4-aminopyridine (Sigma 275875).

### Primary cultures of hippocampal neurons

Hippocampi were dissected out from neonate Balb/c mice brains (0–1 days old). Hippocampi were washed three times in a HBSS-based solution containing: 4 mM NaHCO3, 5 mM HEPES and Hank’s balanced salt solution (Sigma), pH adjusted to 7.3–7.4 at 4°C. Tissues were digested in a solution including: 137 mM NaCl, 5 mM KCl, 7 mM Na2HPO4, 25 mM HEPES, 4.45 mg/ml trypsin type XI (Sigma) and 1614 U/ml DNase type IV (Sigma), pH adjusted to 7.2 at 4°C. Hippocampal tissues were incubated for 10 min in 37°C and washed once with 5 ml HBSS/20% FBS (fetal bovine serum) and once with HBSS. The cells were dissociated in a HBSS solution including 13.15 mM MgSO4 and 1772 U/ml DNase type IV (Sigma). Next, the cells were mechanically triturated with fire-polished Pasteur pipettes. HBSS/20% FBS was added to the dissociated cells and the mixture was centrifuged at 1,000 x *g*, at 4°C for 10 min. The supernatant was discarded and a plating medium including MEM (Gibco), 24.7 mM glucose, 0.089 mg/ml transferrin (Calbiochem), glutaMAX (Gibco), 0.75 U/ml insulin (Sigma), 10% FBS (Biological Industries) and SM1 (StemCell NeuroCult neuronal supplement) was added to the pellet. The cells were resuspended in the plating medium with fire-polished Pasteur pipette and viable cells were counted; 0.5 ml of the cell suspension was added to glass coverslips coated with Matrigel (Corning) in a 24-wells plate, at a density of ∼180,000 cells per well. Two days after plating, 0.5 ml of feeding medium MEM, 26.92 mM glucose, 0.097 mg/ml transferrin, glutaMAX, SM1 and 3 μM cytosine arabinoside [Ara-C] (Sigma) was added to each well. Twice a week, half of the medium was removed from the wells and replaced with the same volume of feeding medium.

### Recombinant AAV-Dlx-mCherry plasmid and infection

To identify GABAergic neurons, we infected hippocampal cultures with a recombinant virus derived from an AAV-viral vector driving the expression of the fluorescent protein mCherry under the control of the specific GABAergic hDlx promoter. Recombinant AAV-virus-Dlx-mCherry plasmid was prepared by inserting the hDlx promoter sequence (541 bp) upstream the coding sequence in the backbone of the pAAV2-mCherry plasmid. The Dlx promoter was shown to restrict reporter expression *in vivo* to all GABAergic interneurons in the forebrain, including hippocampus, as well as in cultured neurons *in vitro* (Dimidschstein et al., 2016; Lezmy et al., 2020). The recombinant AAV2-virus-Dlx-mCherry was produced using standard production methods in HEK 293 cells. All batches produced were in the range of 10^9^ to 10^10^ viral particles per ml. Infections of hippocampal cultures were performed at 6 DIV and recording were carried out at 12–14 DIV.

### Patch-clamp electrophysiology

Patch clamp was performed in the whole-cell configuration. Signals were filtered at 4 kHz and digitized at 10 kHz. All signals were amplified using Multiclamp 700B (Molecular Devices). In the experiments of chronic RTG exposure involving recordings at different time points (4–48 h), untreated cells were also recorded to survey any possible variability, but no particular variance was observed in control untreated cells within the recording time window. For recordings in the current-clamp configuration, the extracellular solution contained 145 mM NaCl, 3 mM KCl, 10 mM HEPES, 15 mM glucose, 1.2 mM MgCl_2_, and 1.8 mM CaCl_2_ (pH was adjusted to 7.4 with NaOH, osmolarity∼315 mOsm). Microelectrodes with resistances of 4–7 MΩ were pulled from borosilicate glass capillaries (Harvard Apparatus) and filled with an intracellular solution. The intracellular solution contained: 135 mM KCl, 1 mM KATP, 1 mM MgATP, 2 mM EGTA, 1.1 mM CaCl_2_, 10 mM HEPES, and 5 mM glucose (pH adjusted to 7.3 with KOH, osmolarity∼300 mOsm). For recording of spontaneous excitatory post-synaptic currents (sEPSCs) and spontaneous inhibitory post-synaptic currents (sIPSCs), the same extracellular solution was used. The reversal potentials for sEPSCs and sIPSCs were experimentally verified prior to the experiments with retigabine with the recording solutions containing, respectively, either 30 μM picrotoxin + 10 μM Bicuculline for sEPSCs or 10 μM NBQX + 10 μM AP5 for sIPSCs. The intracellular solution contained: 0.4 mM GTP, 4 mM Na2ATP, 127 mM CsOH, 127 mM D-gluconic acid, 4 mM CsCl, 10 mM HEPES, 8 mM NaCl, and 0.4 mM EGTA (pH adjusted to 7.25 with CsOH). 5 mM QX314Br was added to the intracellular solution prior to recordings. The sEPSCs and sIPSCs were recorded on the same cell where the voltage was held on the reversal potential of GABA_*A*_ chloride channel (−40 mV) and glutamate receptors (+ 10 mV), respectively. Liquid junction potential was calculated and subtracted from the recorded voltage (+ 5 mV). The reversal potential of GABA_*A*_ chloride channels was depolarized because of the intracellular addition of bromide ions together with QX314 as GABA_*A*_ channel was shown to be permeable to Br^–^. For the experiments inspecting the intrinsic properties of the neurons, synaptic blockers were added to the extracellular solution to prevent spontaneous spikes: 30 μM picrotoxin, 10 μM Bicuculline, 10 μM NBQX, and 10 μM AP-5. For recordings in the voltage-clamp configuration, the extracellular solution contained 140 mM NaCl, 2.5 mM KCl, 5 mM HEPES, 5 mM glucose, 1.2 mM MgCl_2_, and 1.8 mM CaCl_2_ (pH was adjusted to 7.4 with NaOH, osmolarity∼315 mOsm); 1 μM TTX and 0.2 mM 4-aminopyridine were added to the extracellular solution to block voltage-gated Na^+^ currents and I_*A*_ K^+^ currents, respectively. The intracellular solution contained: 130 mM K-gluconate, 6 mM KCl, 2 mM Na_2_ATP, 10 mM HEPES, 1.1 mM EGTA, and 0.1 mM CaCl_2_ (pH adjusted to 7.2.5 with KOH, osmolarity∼300 mOsm). All electrophysiological experiments were performed at room temperature. In the current-clamp configuration, the threshold current was measured by a 2 ms depolarizing current injection in 50 pA increments until a single action potential (AP) was evoked. For evoking a spike train, a 400 ms depolarizing current was injected in 50 pA increments. The firing frequency for each injected current was calculated to produce F-I curves. Action potential amplitude was measured between the RMP and the peak height of the spike. Action potential width was measured as the time between the two points that are exactly half of the AP amplitude. The AHP size was measured as the area above the signal following the spike and the RMP, until the return of the signal to RMP. In the voltage-clamp configuration, cells were held at −60 mV. A step to −20 mV was then given for 6 s, to open M-currents and remove residual inactivating voltage-dependent currents. Then, the voltage was brought back to −60 mV for 4 s to close M-currents, followed by another −20 mV step for 2 s. After leak subtraction, the M-current was calculated by subtracting the peak tail current at −60 mV in the presence of RTG (10 μM), to that in the presence of XE991 (10 μM).

### Immunostaining

Retigabine (RTG) (10 μM) was added to hippocampal neurons for 24 h (for VGlut staining) or 48 h (for VGAT staining) prior to fixation, meaning that the cells (control and treated) were at the same age when fixed for immunostaining (13 DIV). Neurons were fixed in 4% paraformaldehyde for 10 min and washed four times with phosphate buffered saline (PBS). Permeabilization of the membrane was performed by adding 0.1% Triton X-100 (Sigma) in a blocking solution (PBS with 0.1% BSA and 5% goat serum) for 4 min. After washing once with PBS, blocking solution was added to the coverslips for 15 min. Primary antibodies were added, and the neurons were incubated for 1 h at room temperature. The coverslips were washed three times with PBS and incubated at room temperature for 1 h with the secondary antibodies. After washing three times with PBS, the coverslips were mounted in Fluoromount (Sigma). The primary antibodies used for immunostaining were: rabbit α-Kv7.3 (1:150, Alomone Labs), mouse α-AnkyrinG (1:500, Neuromab), mouse α-FGF14 (1:500, Neuromab, 75096), rabbit α-MAP2 (1:1000, Millipore), rabbit α-VGAT (1:500, Synaptic Systems, 131002), guinea pig α-VGlut (1:500, Millipore, AB5905). The secondary antibodies used were: donkey α-mouse Alexa488 (1:1000, Jackson, 715-545-150), donkey α-rabbit Cy3 (1:1000, Jackson, 711-165-152), and donkey α-guinea pig Cy3 (1:1000, Jackson, 706-165-148).

### Data analysis and statistics

For all experiments, data were collected from at least three different batches or more. Control cells were collected from each batch to minimize possible variations between batches. All graphs were built with Prism 9.0 (GraphPad). Error bars represent standard error of the mean (SEM). In acute RTG treatments that were carried out in the same neuron, statistical comparisons between untreated and treated cells were performed using a two-tailed paired *t*-test. When chronic treatments involved more than two independent groups of cells without matching between measures, statistical comparisons were performed using one-way ANOVA and *Post hoc* Dunnett’s Multiple Comparison Test. For evoked spike discharge, statistical comparisons were performed using two-way ANOVA and Bonferonni correction. Analyses of patch-clamp recordings were performed with Clampfit 10.4 (Molecular Devices). Images from immunocytochemistry experiments were obtained using confocal microscopy (Leica STED TCS SP5 II microscope) with oil-immersion objectives of 63x. Images were converted to TIFF files and imported into MATLAB for blind analysis, using a self-written algorithm as previously (Lezmy et al., 2017, 2020). Semi-automatically, the algorithm recognizes the soma and the axon. At each pixel along the axon, fluorescence intensity is measured. AIS was defined to be between the proximal and distal points along the axons, in which the fluorescence intensity was above 50% of the maximum. The AIS’s length and distance from the soma were then calculated and exported to a Microsoft Excel file.

## Results

### Sustained M-channel activation by retigabine induced adaptive changes in intrinsic excitability of cultured excitatory hippocampal neurons

**FIGURE 1:**
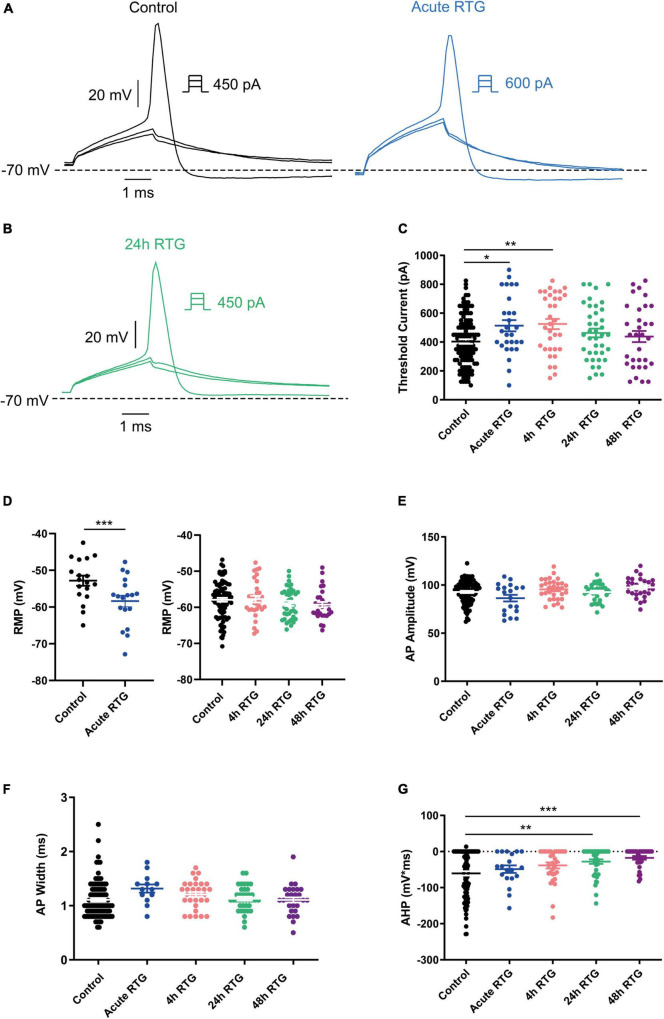
Sustained M-channel activation by retigabine (RTG) induced adaptive changes in intrinsic excitability of excitatory hippocampal neurons. **(A)** Representative traces of solitary spike discharge evoked by injecting depolarizing current for 2 ms with increments of 50 pA, under control conditions (black) and after acute RTG (10 μM) exposure (blue). **(B)** Representative trace of solitary spike discharge evoked by injecting depolarizing current for 2 ms with increments of 25 pA following 24 h RTG exposure. **(C)** Summary of the acute and chronic effects of RTG on the threshold current. Acute and 4 h RTG exposure significantly increased the threshold current compared to control cells (*n* = 121, *n* = 28, *n* = 33, *n* = 39, and *n* = 32 for control, acute, 4, 24, and 48 h RTG treatment, respectively; one-way ANOVA F(4,248) = 3.944; Dunnett’s multiple-comparisons test; *p = 0.0235 and **p = 0.0049, respectively). Following 24 and 48 h of RTG treatment, the threshold current was not significantly different from control cells. **(D)** The RMP was significantly hyperpolarized after acute RTG exposure (*n* = 18, two-tailed paired *t*-test, *t* = 4.414, df = 17, ****p = 0.0004). Following 4, 24, and 48 h of RTG treatment, the resting membrane potential (RMP) was not significantly different from control [*n* = 89, *n* = 31, *n* = 38, and *n* = 30 for control, 4, 24, and 48 h RTG exposure, respectively; one-way ANOVA, F(3,184) = 0.923]. **(E)** The action potential amplitude was not different within the experimental groups (*n* = 107, *n* = 19, *n* = 31, *n* = 38, and *n* = 30 for control, acute, 4, 24, and 48 h RTG exposure, respectively). **(F)** The action potential width was not different within the experimental groups (*n* = 107, *n* = 19, *n* = 31, *n* = 38, and *n* = 30 for control, acute, 4, 24, and 48 h RTG exposure, respectively). **(G)** After 24 and 48 h of chronic RTG exposure, a significant increase in the after-hyperpolarization (AHP) was observed [*n* = 107, untreated control: –61 ± 6 mV*ms; *n* = 38, 24 h: –28 ± 6 mV*ms; *n* = 30, 48 h: –18 ± 5 mV*ms; one-way ANOVA, F(4, 220) = 5.726; Dunnett’s multiple-comparisons test; ** = 0.0039 and ***p = 0.0003, respectively].

**FIGURE 2:**
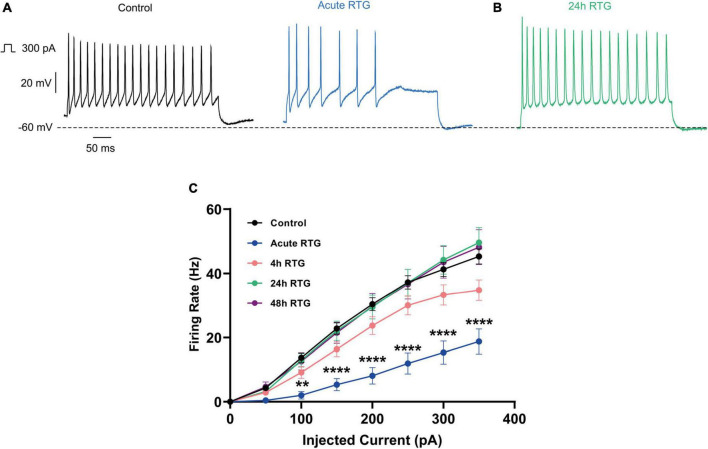
Sustained M-channel activation by retigabine (RTG) induced adaptive changes in the F-I curve of excitatory hippocampal neurons. **(A,B)** Representative traces of spike discharge evoked by 300 pA of depolarizing current injection for 400 ms, under control conditions (A, black), acute RTG exposure (A, blue) and 24 h chronic RTG exposure (B, green). **(C)** The frequency of evoked spike discharge (F-I curves) was significantly reduced after acute RTG exposure, while chronic RTG treatment triggered a homeostatic adaptation, where the values returned already after 4 h close to untreated control conditions [*n* = 29–94; two-way ANOVA treatment F (4, 1720) = 50.73, ****P < 0.0001 and interaction F (28, 1720) = 2.762, ****P < 0.0001].

We have previously shown that sustained M-channel blockade by XE991 concomitantly triggers a fast (1–4 h) adaptation of intrinsic excitability and a distal relocation of Kv7.3 and Nav channels along the AIS in cultured hippocampal neurons (Lezmy et al., 2017, 2020). Here, we asked whether this homeostatic plasticity was bidirectional by investigating the acute and chronic effects of the M-channel opener RTG on hippocampal neuronal excitability. We first examined the effects of RTG on intrinsic excitability of excitatory neurons. GABAergic inhibitory neurons, which represent about 20% of the neuronal culture were identified by infecting hippocampal cultures with a recombinant virus derived from an AAV-viral vector driving the expression of the fluorescent protein mCherry under the control of the specific GABAergic hDlx promoter (Dimidschstein et al., 2016; Lezmy et al., 2020). In contrast, the predominant excitatory neurons were recognized as those that were negative to mCherry fluorescence. Whole-cell patch-clamp recordings from excitatory neurons of hippocampal cultures [12 to 13 days *in vitro* (DIV)] showed that acute RTG (10 μM) exposure reduces the intrinsic excitability, as reflected by a significant increase in the threshold current corresponding to the 2 ms injected depolarizing current necessary to evoke a single spike [Figures 1A,C; *n* = 121, untreated control: 404 ± 16 pA; *n* = 28, acute RTG: 513 ± 39 pA; one-way ANOVA F(4,248) = 3.944; Dunnett’s multiple-comparisons test, *p = 0.0235]. While acute RTG (10 μM) significantly hyperpolarized the resting membrane potential (RMP) (Figure 1D; control = −53 ± 1 mV and acute RTG = −58 ± 2 mV, *n* = 18, two-tailed paired *t*-test, *t* = 4.414, df = 17, ****p = 0.0004), it did not affect the action potential (AP) amplitude and width and the after-hyperpolarization (AHP) (Figures 1E–G). Next, we examined how chronic RTG treatment (4–48 h) affects intrinsic excitability of excitatory neurons. RTG (10 μM) was stable during prolonged incubation conditions, as shown by a significant decrease in the spontaneous firing rate following acute exposure of naive hippocampal neurons to a 10 μM RTG solution, formerly incubated for 48 h with a hippocampal culture (4.2-fold inhibition; *n* = 13, two-tailed paired *t*-test *t* = 3.640, df = 12, **P = 0.0034). Following 4 h exposure to 10 μM RTG, the RMP already exhibited homeostatic adaptation and returned to its initial untreated value (Figure 1D), while the threshold current was compensated only after 24 h of chronic RTG treatment (Figure 1C). After 24 and 48 h of chronic RTG exposure, a significant decrease in the AHP was observed [Figure 1G; *n* = 107, untreated control: −61 ± 6 mV*ms; *n* = 38, 24 h: −28 ± 6 mV*ms; *n* = 30, 48 h: −18 ± 5 mV*ms; one-way ANOVA, F(4, 220) = 5.726; Dunnett’s multiple-comparisons test; **p = 0.0039 and ***p = 0.0003, respectively]. In line with the results of the threshold current, the frequency of evoked spike discharge (F-I curves) was significantly reduced after acute RTG exposure; likewise, chronic RTG treatment triggered an homeostatic adaptation, where the values returned already after 4 h close to untreated conditions [Figures 2A–C; *n* = 29–94; two-way ANOVA treatment F (4, 1720) = 50.73, ****P < 0.0001 and interaction F (28, 1720) = 2.762, ****P < 0.0001].

### Chronic M-channel activation of excitatory neurons triggered a slow homeostasis of the spontaneous firing rate and post-synaptic currents but did not alter the excitation-to-inhibition ratio

**FIGURE 3:**
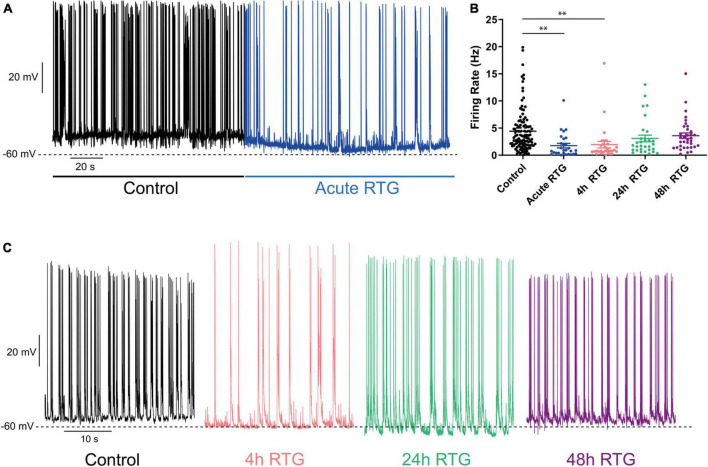
Chronic M-channel activation of excitatory neurons by retigabine (RTG) triggered a slow homeostasis of the spontaneous firing rate. **(A)** Representative trace of spontaneous spiking activity of a neuron before (black) after acute RTG exposure (blue). **(B)** Summary of the acute and chronic effects of RTG on the spontaneous spiking activity. A significant decrease in the ongoing firing rate was found after acute RTG exposure (2.5-fold decrease) and was still observed following 4 h of RTG chronic treatment [2.3-fold decrease, one-way ANOVA, F(4, 235) = 5.314; Dunnett’s multiple-comparisons test, *n* = 118 untreated, *n* = 28, **P = 0.0015 and *n* = 29, **P = 0.0030, for acute and 4 h RTG exposure, respectively]. At longer RTG exposure times (24 and 48 h), the ongoing firing rate progressively increased and returned to the values of untreated neurons. **(C)** Representative traces showing that while after 4 h (pink) of RTG treatment the firing rate is decreased, the spontaneous activity after chronic 24 h (green) and 48 h (purple) RTG exposure is similar to that of an untreated neuron (black).

**FIGURE 4:**
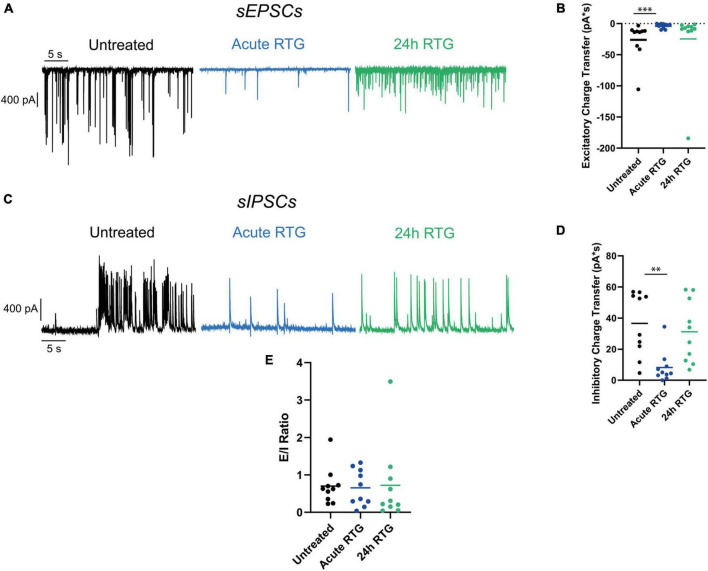
Chronic M-channel activation of excitatory neurons triggered a homeostasis of both spontaneous excitatory post-synaptic currents (sEPSCs) and spontaneous inhibitory post-synaptic currents (sIPSCs) but did not alter the excitatory/inhibitory (E/I) ratio. **(A)** Representative traces showing sEPSCs of control (black trace), acute retigabine (RTG) (blue trace) and 24 h RTG (green trace) treatments. **(B)** Acute RTG treatment promoted a significant decrease in sEPSCs charge transfer (*n* = 10, for sEPSCs mean control: –26.0 pA*s, mean acute RTG: –3.8 pA*s; Kruskal-Wallis test: ***P = 0.0009), while following 24 h RTG exposure it returned to values similar to those of untreated neurons (*n* = 10, mean 24 h RTG: 24.6 pA*s). **(C)** Representative traces showing sIPSCs of control (black trace), acute RTG (blue trace), and 24 h RTG (green trace) treatments. **(D)** Acute RTG treatment promoted a significant decrease in sIPSCs charge transfer (*n* = 10; mean control: 36.7 pA*s, mean acute RTG: 8.2 pA*s; Kruskal-Wallis test: **P = 0.0023) while following 24 h RTG exposure it returned to values similar to those of untreated neurons (*n* = 10, mean 24 h RTG: 31.2 pA*s). **(E)** E/I ratio was calculated by dividing sEPSCs by sIPSCs charge transfers of the same neuron. The E/I ratio was not significantly affected by acute RTG exposure (*n* = 10; E/I ratio control: 0.70 and E/I ratio acute RTG: 0.65; Kruskal-Wallis test, *P* = 0.5092) as well as by 24 h RTG exposure (24 h RTG: 0.72).

Next, we examined how sustained M-channel activation by RTG affects the spontaneous ongoing firing rate of hippocampal neurons. A significant decrease in the ongoing firing rate was found after acute RTG exposure (Figures 3A–C; 2.5-fold decrease) and was still observed following 4 h of RTG chronic treatment [2.3-fold decrease, one-way ANOVA, F(4, 235) = 5.314; Dunnett’s multiple-comparisons test, *n* = 118 untreated, *n* = 28, **P = 0.0015 and *n* = 29, **P = 0.0030, for acute and 4 h RTG exposure, respectively] (Figures 3A–C). At longer RTG exposure times (24 and 48 h), the ongoing firing rate progressively increased and returned to the values of untreated neurons, reflecting a slow homeostatic plasticity process (Figures 3B,C; one-way ANOVA *n* = 30, *P* = 0.2228 and *n* = 35, *P* = 0.6194, for 24 and 48 h RTG treatment, respectively). To examine the potential alterations in the synaptic output of the network, spontaneous excitatory post-synaptic currents (sEPSCs) and spontaneous inhibitory post-synaptic currents (sIPSCs) were recorded from excitatory hippocampal neurons by holding the same cell successively at the inhibitory and excitatory reversal potentials, respectively (see Methods and Figure 4). Acute RTG treatment promoted a significant decrease in both sEPSCs and sIPSCs charge transfer (*n* = 10, for sEPSCs mean control: −26.0 pA*s, mean acute RTG: −3.8 pA*s; Kruskal-Wallis test: ***P = 0.0009 and for sIPSCs mean control: 36.7 pA*s, mean acute RTG: 8.2 pA*s; Kruskal-Wallis test: **P = 0.0023) (Figures 4A–D). Thus, the excitatory/inhibitory (E/I) ratio was not significantly affected by acute RTG exposure (*n* = 10; E/I ratio control: 0.70 and E/I ratio acute RTG: 0.65; Kruskal-Wallis test, *P* = 0.5092) (Figure 4E). Following 24 h of RTG exposure, both sEPSCs and sIPSCs charge transfer underwent homeostatic compensation and returned to values similar to those of untreated neurons (Figures 4A–D; *n* = 10, for sEPSCs mean 24 h RTG: 24.6 pA*s and for sIPSCs mean 24 h RTG: 31.2 pA*s). Therefore, the E/I ratios were unchanged by chronic RTG treatment and remained similar to those control and acute RTG exposure (Figure 4E; E/I ratio 24 h RTG: 0.72).

### The homeostatic adaptation to retigabine treatment of excitatory neurons was concomitant to a proximal axon initial segment relocation of Kv7.3, Ankyrin G and FGF14 as well as a decrease in M-current density

**FIGURE 5:**
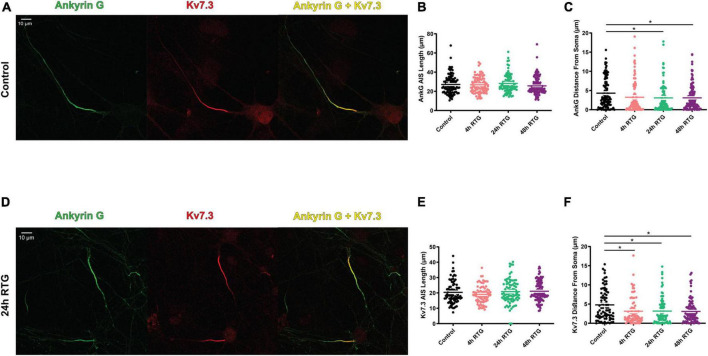
Chronic retigabine (RTG) treatment of excitatory neurons triggered a proximal axon initial segment (AIS) relocation of Kv7.3 and Ankyrin G. **(A)** Representative images of excitatory neurons from control neurons stained with anti-ankyrin G (green) and anti-Kv7.3 (red) antibodies and merged (yellow). **(B)** Chronic RTG treatment did not affect the length of the ankyrin G segment. **(C)** Ankyrin G significantly relocated proximally to the soma by about 2 μm after 24 h and 48 h of RTG exposure [one-way ANOVA, F (3, 438) = 2.995; Dunnett’s multiple-comparisons test, *n* = 107 for control, *n* = 99 *P* = 0.0850, *n* = 98 *P = 0.0369 and *n* = 138 *P = 0.0224 for 4, 24, and 48 h RTG treatment, respectively]. **(D)** Representative images of excitatory neurons from neurons treated for 24 h with RTG and stained with anti-ankyrin G (green) and anti-Kv7.3 (red) antibodies and merged (yellow). **(E)** Chronic RTG treatment did not affect the length of the Kv7.3 segment. **(F)** The distance from the soma of the Kv7.3 segment became significantly shorter by about 2 μm following 4 h, 24 h and 48 h of RTG treatment [one-way ANOVA, F (3, 354) = 5.136; Dunnett’s multiple-comparisons test, *n* = 83 for control, *n* = 78 **P = 0.0058, *n* = 89 **P = 0.0051 and *n* = 108 **P = 0.0019 for 4, 24, and 48 h RTG treatment, respectively].

**FIGURE 6:**
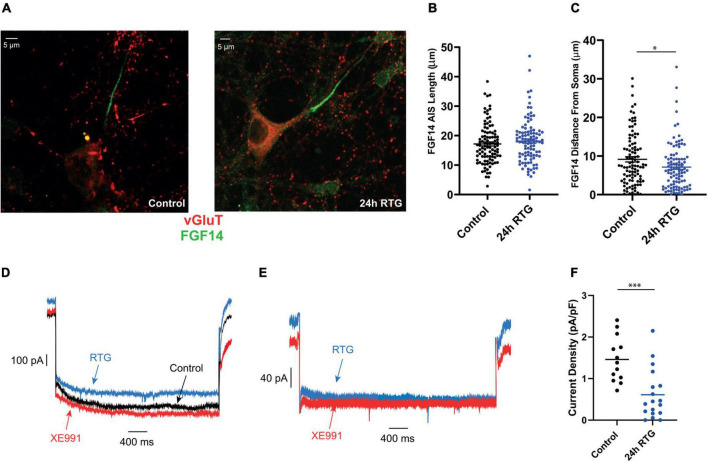
Chronic retigabine (RTG) treatment of excitatory neurons triggered a proximal axon initial segment (AIS) relocation of FGF 14 and a decrease in M-current density. **(A)** Representative images of excitatory neurons from control (left) and 24 h RTG treated neurons (right), stained with anti-vGluT (red), and anti-FGF14 (green) antibodies. **(B)** The length of the FGF14 segment of control and 24 h RTG treated neurons are not significantly different. **(C)** The distance from the soma of the FGF14 segment became significantly shorter by about 2 μm following 24 h RTG treatment (unpaired two-tailed *t*-test, *t* = 2.238, df = 200, *n* = 101, *P = 0.0263). **(D)** Representative trace of the M-current of the same control neuron before (black) and after acute exposure to RTG (10 μM) (blue), followed by XE991 (10 μM) (red). M-currents were recorded using the classical deactivation tail protocol (Adams et al., 1982), where cells were held at –60 mV. A step to –20 mV was then given for 6 s, to open M-currents and remove residual inactivating voltage-dependent currents. Then, the voltage was brought to –60 mV for 4 s to close M-currents, followed by another –20 mV step for 2 s. **(E)** Representative trace of the M-current recorded after 24 h of chronic RTG treatment (blue) and then following acute XE991 exposure (red). **(F)** Following 24 h RTG exposure, the current density was significantly decreased by 58% (1.46 pA/pF and 0.61 pA/pF for control and 24 h RTG, respectively; *n* = 18/13, two-tailed Mann Whitney test, ***p = 0.0003).

We previously showed that chronic neuronal hyperactivity, induced by M-channel inhibition of cultured hippocampal excitatory neurons, triggered a fast (1–4 h) intrinsic homeostatic plasticity that was paralleled by a distal relocation of Kv7.3, ankyrin G and FGF14 along the AIS (Lezmy et al., 2017, 2020). Here, we examined the location of Kv7.3 and ankyrin G at the AIS of excitatory neurons by immnocytofluorescence, using specific antibodies. Results show that the distance from the soma of the Kv7.3 segment became significantly shorter by about 2 μm following 4, 24, and 48 h of RTG treatment [Figures 5A–F; one-way ANOVA, F (3, 354) = 5.136; Dunnett’s multiple-comparisons test, *n* = 83 for control, *n* = 78 **P = 0.0058, *n* = 89 **P = 0.0051 and *n* = 108 **P = 0.0019 for 4, 24, and 48 h RTG treatment, respectively]. Ankyrin G also relocates proximally by about 2 μm only after 24 and 48 h of RTG exposure [Figures 5A–F; one-way ANOVA, F (3, 438) = 2.995; Dunnett’s multiple-comparisons test, *n* = 107 for control, *n* = 99 *P* = 0.0850, *n* = 98 *P = 0.0369 and *n* = 138 *P = 0.0224 for 4, 24, and 48 h RTG treatment, respectively]. The total length of Kv7.3 and ankyrin G segments was unchanged (Figures 5A–F). FGF14 is a protein physically bridging Nav1.6 and Kv7.2/channels along the AIS and represents therefore a valuable reporter of Nav1.6 and Kv7.2/3 channels location at the AIS (Pablo and Pitt, 2017). Thus, we examined the location of FGF14 at the AIS in excitatory neurons by double immunocytofluorescence using antibodies against FGF14 and the vesicular glutamate transporter 1, vGLUT1 (Figures 6A–C). FGF14 location was inspected after 24 h RTG exposure, the time by which homeostatic adaptation was fully reached (see Figures 1–3). Remarkably and similar to Kv7.3 and ankyrin G segments, the data indicate that the distance from the soma of the FGF14 segment became significantly shorter by about 2 μm following 24 h RTG treatment (Figures 6A–C; unpaired two-tailed *t*-test, *t* = 2.238, df = 200, *n* = 101, *P = 0.0263). The total length of the FGF14 label was unchanged (Figure 6B). Since the homeostatic shortening of the Kv7.3, ankyrin G and FGF14 distances from the soma, was quite small, though significant, we explored whether other factors such the M-current density, could contribute to the compensatory increase in excitability. M-currents were recorded using the classical tail protocol (Adams et al., 1982) and data showed that following 24 h RTG exposure, the current density was significantly decreased by 58% (Figures 6D–F; 1.46 pA/pF and 0.61 pA/pF for control and 24 h RTG, respectively; *n* = 18/13, two-tailed Mann Whitney test, ***p = 0.0003).

### Inhibitory hippocampal neurons exhibited poor intrinsic homeostatic plasticity following M-channel activation by retigabine

**FIGURE 7:**
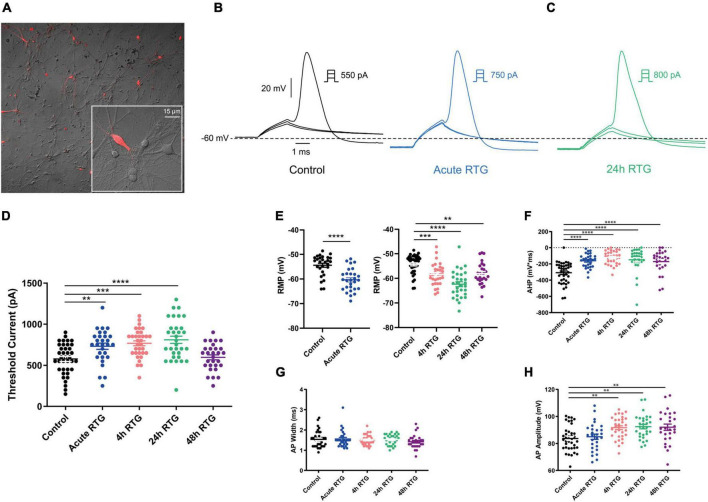
Effects of sustained M-channel activation by retigabine (RTG) on intrinsic excitability of GABAergic inhibitory hippocampal neurons. **(A)** To identify GABAergic neurons, cultured hippocampal neurons were infected with a recombinant virus derived from an AAV-viral vector driving the expression of the fluorescent protein mCherry under the control of the specific GABAergic hDlx promoter. **(B,C)** Representative traces of solitary spike discharge evoked by injecting depolarizing current for 2 ms, with increments of 50 pA, under control conditions (B, black), after acute RTG exposure (B, blue) and after 24 h RTG exposure (C, green). **(D)** Following acute, 4 and 24 h of RTG exposure, there is a significant increase in the threshold current for firing a solitary spike compared with controls [one-way ANOVA; F(4, 156) = 9.196; Dunnett’s multiple-comparisons test; *n* = 29, **p = 0.007, *n* = 33, ***p = 0.0003, *n* = 32, ****p < 0.0001, and *n* = 33, respectively]. After 48 h of RTG treatment, the threshold current was similar to that of controls (*n* = 29). **(E)** The resting membrane potential was significantly hyperpolarized after acute RTG exposure (left, two-tailed paired *t*-test, *t* = 8.400, *n* = 28, df = 27, ****p < 0.0001), and remained hyperpolarized after 4, 24, and 48 h of RTG exposure compared to control [right, one-way ANOVA, F(3,123) = 18.23; Dunnett’s multiple-comparisons test; *n* = 32, ***p = 0.0006, *n* = 30, ****p < 0.0001, *n* = 27, **p = 0.0038, and *n* = 38, respectively]. **(F)** Following acute, 4, 24, and 48 h of RTG treatment, after-hyperpolarization (AHP) was larger than in control cells [one-way ANOVA; F(4, 150) = 13.44; Dunnett’s multiple-comparisons test; ****p < 0.0001, *n* = 28, *n* = 32, *n* = 30, *n* = 27, and *n* = 38, respectively]. **(G)** No significant changes in spike width were observed following RTG treatment within all experimental groups. **(H)** Spike amplitude was larger in cells exposed to RTG for 4, 24, and 48 h, compared to untreated cells [one-way ANOVA; F(4, 150) = 6.257, Dunnett’s multiple-comparisons test; *n* = 32, **p = 0.0029, *n* = 30, **p = 0.0012, *n* = 27, **p = 0.0031, *n* = 38, and *n* = 28, respectively].

**FIGURE 8:**
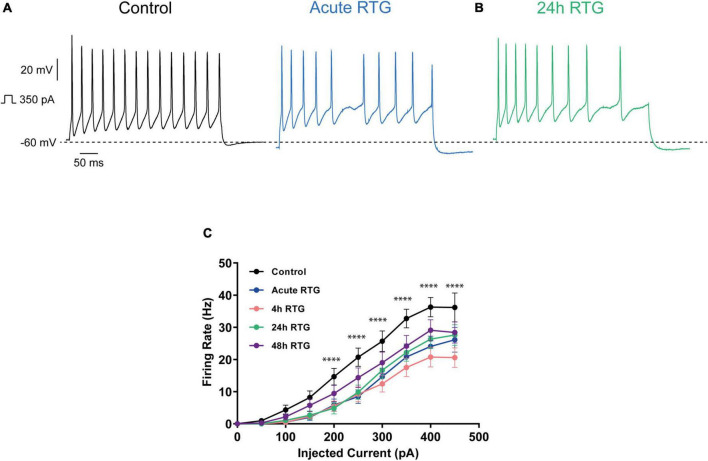
Effects of sustained M-channel activation of GABAergic inhibitory hippocampal neurons by retigabine (RTG) on the F-I curve. **(A,B)** Representative traces of spike discharge evoked by 350 pA of depolarizing current injection for 400 ms, under control conditions (A, black), acute RTG exposure (A, blue) and 24 h chronic RTG exposure (B, green). **(C)** The frequency of evoked spike discharge was significantly reduced following acute, 4 and 24 h of RTG exposure for injected depolarizing currents of 200 pA and above [*n* = 21–37; two-way ANOVA treatment F (4, 1500) = 27.02, ****P < 0.0001 and interaction F (36, 1500) = 1.261, *P* = 0.1393]. At 48 h RTG exposure, the frequency of spikes was not significantly different from control untreated neurons.

We examined the effects of RTG on intrinsic excitability of GABAergic inhibitory neurons, which were identified by the fluorescent protein mCherry under the control of the specific GABAergic hDlx promoter (Dimidschstein et al., 2016; Figure 7A). Similar to excitatory neurons, acute RTG (10 μM) exposure reduced the intrinsic excitability, as mirrored by a significant increase in the threshold current [Figures 7B,D; *n* = 38, untreated control: 578 ± 31 pA; *n* = 29, acute RTG: 731 ± 38 pA; one-way ANOVA F(4, 156) = 9.196; Dunnett’s multiple-comparisons test, **P = 0.007]. Acute RTG also hyperpolarized the RMP (two-tailed paired *t*-test, *t* = 8.400, *n* = 28, df = 27, ****p < 0.0001), decreased the AHP [one-way ANOVA; F(4, 150) = 13.44; Dunnett’s multiple-comparisons test; ****p < 0.0001, *n* = 28] and the AP amplitude but did not affect the AP width (Figures 7E–H). No homeostatic adaptation for the threshold current was observed at 4 and 24 h of RTG exposure. Only after 48 h RTG treatment, the threshold current decreased close to the untreated value [one-way ANOVA; F (4, 156) = 9.196; Dunnett’s multiple-comparisons test; *n* = 33, ***P = 0.0003, *n* = 32, ****P < 0.0001 and *n* = 29, *P* = 0.9820, respectively]. Similarly, no homeostatic compensation was found for the RMP, the AHP and the AP amplitude at any time of RTG chronic treatment (Figures 7E–H). The frequency of evoked spike discharge (F-I curves) was significantly reduced after acute RTG exposure for injected depolarizing currents of 200 pA and above (Figures 8A–C); no homeostatic plasticity was obtained at 4 and 24 h of chronic RTG treatment, where the frequency of spike discharge was still significantly reduced compared to untreated neurons. Similar for the threshold current, only after 48 h RTG treatment, a poor homeostatic adaptation was observed, with the frequency values returning close to untreated conditions [Figures 8A–C; *n* = 20–37; two-way ANOVA treatment F (4, 1500) = 27.02, ****P < 0.0001 and interaction F (36, 1500) = 1.261, *P* = 0.1393].

### Chronic M-channel activation of inhibitory neurons failed to trigger homeostasis of the spontaneous firing rate and did not affect the length nor the location of FGF14 along the axon initial segment

**FIGURE 9:**
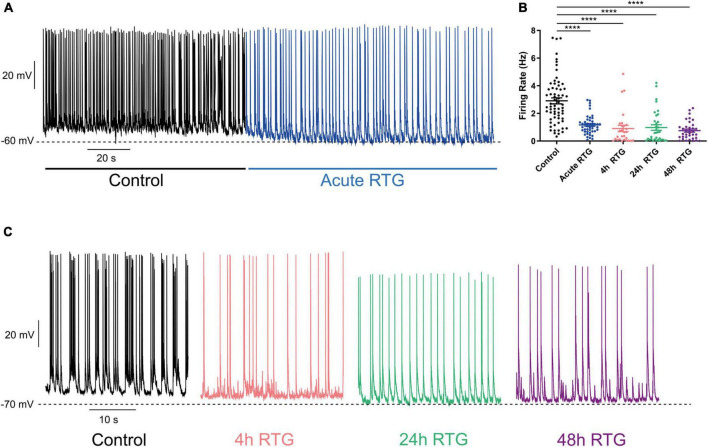
Chronic M-channel activation of inhibitory neurons by retigabine (RTG) failed to trigger homeostasis of the spontaneous firing rate. **(A)** Representative trace of spontaneous activity of a neuron before (black) after acute RTG administration (blue). **(B)** Spontaneous mean firing rate was significantly reduced following acute, 4, 24, and 48 h RTG exposure as compared to control [one-way ANOVA, F(4, 203) = 27.78; Dunnett’s multiple-comparisons test, **** < 0.0001, *n* = 46, *n* = 28, *n* = 34, *n* = 35, and *n* = 65, respectively]. **(C)** Representative traces showing that following 4 h (pink), 24 h (green), and 48 h (purple) of RTG treatment, the spontaneous firing rate was depressed, compared to that of an untreated neuron (black).

**FIGURE 10:**
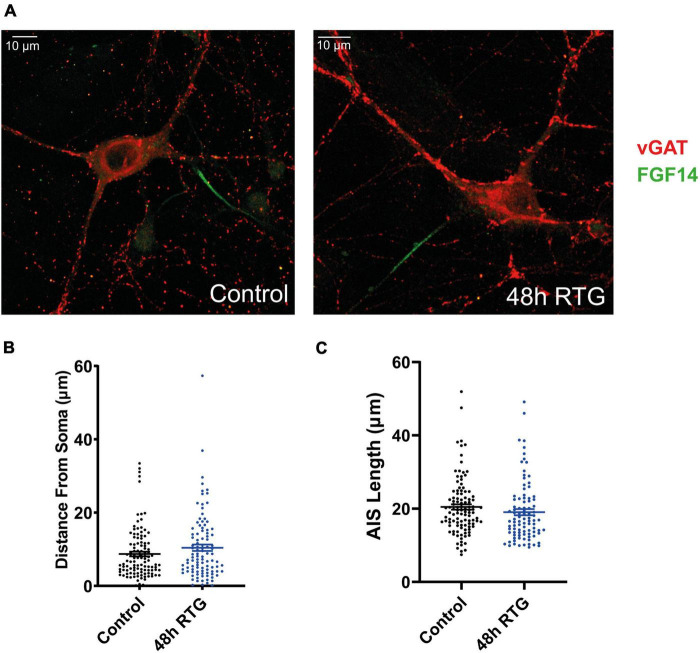
Chronic M-channel activation by retigabine (RTG) did not affect the length nor the location of FGF14 along the axon initial segment (AIS). **(A)** Representative images of an inhibitory untreated neuron (left) and a neuron treated for 48 h with RTG (right). Cells were stained with anti-vGAT (red) and anti-FGF14 (green) antibodies. **(B,C)** The distance from the soma and the length of the FGF14 segment along the AIS in neurons exposed to RTG for 48 h are not significantly different from those of untreated neurons.

Then, we explored how prolonged M-channel activation by RTG affects the spontaneous firing rate of inhibitory hippocampal neurons. In contrast to excitatory neurons, no homeostatic compensation was found for the spontaneous firing rate at any time of RTG chronic treatment. The firing rate remained significantly reduced after acute, 4, 24, and 48 h of drug exposure as compared to untreated control [Figures 9A–C; one-way ANOVA, F(4, 203) = 27.78; Dunnett’s multiple-comparisons test, **** < 0.0001, *n* = 46, *n* = 28, *n* = 34, *n* = 35, and *n* = 65, respectively]. Next, we examined the location of FG14 along the AIS in inhibitory neurons by double immunocytofluorescence using antibodies against FG14 and the vesicular GABA transporter, vGAT (Figures 10A–C). FGF14 location was inspected after 48 h RTG, the time at which weak homeostatic adaptation was found for the threshold current and for the frequency of evoked spike discharge (see Figures 7, 8). Results indicate that the distance from the soma of the FGF14 segment remained unchanged following 48 h RTG treatment (Figures 10A,B; unpaired two-tailed *t*-test, *t* = 1.584, df = 211, *n* = 102–111, *P* = 0.1146). Similarly, the total length of the FG14 label remained unaffected (Figures 10A,C; unpaired two-tailed *t*-test, *t* = 1.265, df = 199, *P* = 0.2074).

## Discussion

Bidirectional homeostasis is essential for normal brain function as it can prevent deleterious states of epileptic activity or quiescence by providing negative feedback control. A wide spectrum of studies has explored bidirectional homeostatic plasticity both *in vitro* and *in vivo* using various pharmacological manipulations of activity or behavioral paradigms. For example, many investigations employed tetrodotoxin, excitatory synaptic blockers or GABA_B_ receptor agonists to chronically block network activity in cortical or hippocampal cultures *in vitro* (Abbott and Nelson, 2000; Burrone et al., 2002; Turrigiano, 2011, 2012; Slomowitz et al., 2015; Vertkin et al., 2015; Turrigiano, 2017). Alternatively, optogenetics, high external potassium or GABA_A_ channel blockers were used to persistently enhance neuronal excitability (Grubb and Burrone, 2010; Yoshimura and Rasband, 2014; Vertkin et al., 2015; Kole and Brette, 2018). *In vivo*, sensory deprivation paradigms such as monocular deprivation, focal retinal lesions, cochlea removal or deprivation of the whisker-to-barrel pathway were applied to depress sensory inputs while behavioral settings like enriched environment were used to increase neuronal activity (Hensch et al., 1998; Kuba et al., 2010; Chen et al., 2011; Barnes et al., 2015; Fu et al., 2015; Milshtein-Parush et al., 2017; Jamann et al., 2021). Although these experiments provided precious knowledge about homeostatic plasticity mechanisms, they rarely explored in the same cellular system the bidirectionality of the plasticity or the impact of the bidirectional perturbation simultaneously on excitatory and inhibitory neurons.

In our *in vitro* studies, we bidirectionally induced the plasticity using the same molecular trigger, the M-channel, in the same hippocampal culture system. M-channels are assembled as heterotetramers of Kv7.2 or Kv7.5 and Kv7.3 subunits. They generate subthreshold, non-inactivating voltage-gated K^+^ currents that play an important role in controlling neuronal excitability and plasticity (Jentsch, 2000; Shah et al., 2002; Delmas and Brown, 2005; Lezmy et al., 2017). M-channels are expressed in both pyramidal neurons and GABAergic interneurons (Cooper et al., 2001). In pyramidal neurons, they primarily control spike frequency adaptation (Peters et al., 2005; Soh et al., 2014), while they regulate the firing pattern of inhibitory neurons in a cell-type specific manner (Soh et al., 2018). Hence, we examined the bidirectionality of homeostatic plasticity induced by M-channel modulation in both excitatory and inhibitory neurons of primary hippocampal cultures. Recently, we showed that chronic neuronal hyperactivity, induced by sustained M-channel blockade with XE991 concomitantly triggered a fast (1–4 h) adaptation of intrinsic excitability and a distal relocation of Kv7.3, Nav channels and FGF14 along the AIS in hippocampal neurons (Lezmy et al., 2017). In contrast, synaptic homeostatic plasticity occurred at a slower timescale (≈ 2 days) and involved decreases in mEPSC amplitude (Lezmy et al., 2020). However, no homeostatic plasticity of intrinsic excitability and network MFR was observed in hippocampal GABAergic neurons, which remained hyperexcitable following chronic M-channel blockage (Lezmy et al., 2020).

In the present study, we examined whether the homeostatic plasticity induced by M-channel inhibition was bidirectional by investigating the acute and chronic effects of M-channel activation on hippocampal neuronal excitability using the opener RTG. We found that persistent M-channel activation by RTG induced adaptive changes in intrinsic excitability of excitatory hippocampal neurons. While acute RTG exposure markedly reduced intrinsic excitability, 4 h exposure to the opener, already produced homeostatic adaptation of the RMP and of the F-I curves, which returned to their initial untreated values. However, the threshold current (or rheobase current) was compensated only after 24 h of chronic RTG treatment. Comparing these data with those obtained for the chronic M-channel blockade by XE991 (Lezmy et al., 2017), we conclude that M-channel modulation triggered a bidirectional homeostatic plasticity in hippocampal excitatory neurons. However, the kinetics of homeostatic compensation were somewhat slower when plasticity was induced by M-channel activation than by M-channel inhibition, especially considering the threshold current, which was adapted already after 1 h of chronic M-channel block (XE991) and only after 24 h of M-channel activation (RTG) (Lezmy et al., 2017). Another striking difference was observed for the AHP that remained significantly small and did not undergo homeostatic compensation following chronic RTG application. Indeed, M-channels contribute to AHP, which limits repetitive firing following bursts of action potentials (Storm, 1989; Tzingounis and Nicoll, 2008). The persistent decrease in AHP observed upon chronic RTG treatment can arise from the compensatory reduction in M-current density (see below). Decreased AHP could also result from a homeostatic reduction in big conductance (BK, KCa1.1) or small conductance calcium-dependent (SK) potassium channels, which also contribute to AHP in hippocampal neurons (Storm, 1989). Like for the persistent M-channel block, chronic M-channel activation of excitatory neurons triggered a slow homeostasis of the spontaneous MFR at a 24–48 h timescale, which suggests that the relatively fast intrinsic adaptation triggered by M-channel modulation is not sufficient to account for the slow homeostatic normalization of the MFR. In contrast to the chronic M-channel blockade by XE991 (Lezmy et al., 2017), chronic RTG exposure triggered homeostatic compensation of both sEPSCs and sIPSCs charge transfer, thereby generating an unchanged E/I ratio.

We previously showed that chronic M-channel inhibition of hippocampal excitatory neurons by XE991, triggered a fast (1–4 h) intrinsic homeostatic plasticity that was paralleled by a distal relocation of Kv7.3, ankyrin G, and FGF14 along the AIS (Lezmy et al., 2017, 2020). Here, the homeostatic adaptation of excitatory neurons to RTG treatment was concomitant to a small but significant proximal AIS relocation of Kv7.3, Ankyrin G and FG14 as well as to a decrease in M-current density. Strategically located between the somatodendritic and axonal compartments, the AIS triggers and shapes action potentials and determines neuronal output. The AIS consists of ion channels and cell adhesion molecules anchored to specialized cytoskeletal protein scaffolds, ankyrin G and βIV spectrin that are connected to actin filaments and microtubules. Actin rings are regularly found (190 nm period) along the AIS and distal axon. Ankyrin G binds subtypes of voltage-gated sodium (Nav) and potassium (Kv) channels *via* a specific analogous motif, ensuring their clustering at the AIS (Grubb et al., 2011; Yoshimura and Rasband, 2014; Adachi et al., 2015; Petersen et al., 2016; Yamada and Kuba, 2016; Kole and Brette, 2018). Several lines of evidence suggest that the AIS dynamically adapts its length and/or location along the axon relative to the soma to modulate the neuronal input/output curve (Grubb and Burrone, 2010; Kuba et al., 2010; Evans et al., 2015; Wefelmeyer et al., 2016; Kole and Brette, 2018). In models with AIS containing both Na^+^ and M-channels, the effect of Nav and Kv7 proximal relocation is expected to enhance excitability (Lezmy et al., 2017). Although small (2 μm), we believe that the proximal AIS relocation of Kv7.3, Ankyrin G and FG14 contributes to a homeostatic increase in neuronal excitability. However, this proximal AIS relocation is probably not enough to provide the necessary homeostatic hyperexcitability and is accompanied by a parallel decrease in M-type K^+^ current density. Many lines of evidence indicate that M-channel activity is highly plastic (Baculis et al., 2020). Triggering epileptic activity in hippocampal neurons increases the expression of Kv7.2 and Kv7.3 subunit transcripts suggesting a homeostatic mechanism to normalize activity levels (Zhang and Shapiro, 2012). Conversely, chronic depression of network activity achieved by NMDA receptor blockers reduces M-currents in dissociated hippocampal neurons (Lee et al., 2015), showing that M-channels appear to modulate their expression bidirectionally in a homeostatic manner. It is worth noting that we did not observe a reciprocal increase in M-current density when we chronically blocked M-channels by XE991 (Lezmy et al., 2017). If we compare these AIS relocation data with those obtained previously for the sustained M-channel blockade by XE991, we conclude that in hippocampal excitatory neurons, there is a bidirectional homeostatic plasticity triggered by M-channel modulation. However, this homeostatic bidirectionality is not symmetric and it mobilizes additional mechanisms such as those involved in the down-regulation of M-currents. Along this line, a recent work reported a temporally asymmetric, bidirectional, activity-dependent remodeling of the AIS and input-output properties of the mouse barrel cortex (Jamann et al., 2021). Deprivation of the whisker-to-barrel pathway for 15 days or longer caused long-term AIS elongation in primary sensory cortex neurons, accompanied by an increase in pyramidal neuron excitability. On the other hand, increasing the activity of the same neuronal population for just 1–3 h *via* exposure of the animals to an enriched environment resulted in AIS shortening and reduced pyramidal neuron output (Jamann et al., 2021).

What mechanisms can account for the decrease in M-currents? A recent study showed that prolonged activity blockade in cultured hippocampal neurons reduces the activity of extracellular signal regulated kinase 1/2 (ERK1/2) followed by a decrease in the activation of brain-derived neurotrophic factor (BDNF) receptor, TrkB, as well as by a reduction in the density of Kv7.3 and ankyrin-G at the AIS (Baculis et al., 2022). It was also recently revealed that a conserved AIS endocytic clearance mechanism plays a crucial role in regulating the levels of transmembrane signaling proteins in the AIS (Eichel et al., 2022), suggesting that these processes may be involved in the decrease of M-currents following chronic RTG exposure.

In contrast to hippocampal excitatory neurons, we found that GABAergic inhibitory neurons barely exhibit intrinsic homeostatic plasticity following sustained M-channel activation by RTG. We also showed that chronic M-channel activation in inhibitory neurons failed to trigger homeostasis of the spontaneous firing rate and did not induce homeostatic plasticity of the AIS. In fact, GABAergic neurons exhibited a persistent hypoexcitability in the continuous presence of RTG. In a way, this feature is the reciprocal picture of GABAergic hippocampal neurons chronically exposed to the M-channel blocker XE991 (Lezmy et al., 2020). It is interesting to notice that a recent *in vivo* work performed in the visual cortex of adult mice showed that following monocular enucleation, inhibitory neurons either become completely inactive or are unable to homeostatically recover their reduced activity (Barnes et al., 2015). On the other hand, another work showed that olfactory bulb inhibitory neurons exhibited a bidirectional inverted AIS plasticity that was dependent on the activity of L-type Ca^2+^ channels (Chand et al., 2015). Why inhibitory neurons in cortical and hippocampal networks are unable to display homeostatic compensations in face of persistent hypo-or hyper-excitability? Given the different types of inhibitory neurons and their various synaptic targets, they may play different roles in homeostatic recovery. It was suggested that excitatory neurons recover in a subnetwork specific manner and that recovery of activity is facilitated by reduced synaptic inhibition (Barnes et al., 2015). Importantly, a substantial subpopulation of inhibitory neurons (e.g., VIP interneurons) has long been recognized as a potential disinhibitory circuit in the hippocampus. In this context, the persistence of inhibitory neuron activity may be necessary to stabilize the network and a recent model study revealed strong inhibitory-to-inhibitory connections underlying a disinhibitory microcircuit as a critical component for stable temporal dynamics and working memory (Kim and Sejnowski, 2021).

In conclusion, our data indicate that in hippocampal excitatory neurons the persistent bidirectional modulation of M-channels triggered a homeostatic plasticity, though not symmetric in terms of timescale and mechanisms. In contrast, M-channels failed to trigger homeostasis of intrinsic plasticity and network firing rate in GABAergic inhibitory neurons.

## Data availability statement

The original contributions presented in this study are included in the article/supplementary material, further inquiries can be directed to the corresponding author.

## Ethics statement

The animal study was reviewed and approved by all experimental protocols conformed to the guidelines of the Institutional Animal Care and Use Committee of Tel-Aviv University, Israel, and to the guidelines of the NIH (animal welfare authorization number: 01-16-012).

## Author contributions

BA designed the research and wrote the manuscript. LB, LS, and JL performed the research and analyzed data. AP analyzed data. All authors contributed to the article and approved the submitted version.
